# Analysis of the dynamic relationship between urban economic growth and energy consumption and environmental quality response from the perspective of an improved VAR model

**DOI:** 10.1371/journal.pone.0348019

**Published:** 2026-05-04

**Authors:** Lei Xie, Weiran Zhou

**Affiliations:** Xinyang Vocational and Technical College, Xinyang, China; University of Jinan, CHINA

## Abstract

To acquire a thorough comprehension of the changing interplay between urban economic expansion and energy usage, the research conducts a dynamic correlation analysis between energy and the economy, integrating heteroskedasticity and differential evolution algorithms to bolster the vector autoregressive model. The experimental results indicated that the improvement strategy enhanced the convergence and solution set distribution of the differential evolutionary algorithm. Granger causality has been identified among urbanization, economic growth, energy consumption, and environmental quality, indicating that timely modifications to economic development and energy strategies can yield positive impacts on urban planning, ecological health, and overall economic progress. While energy consumption and economic growth exhibit a reciprocal influence, it is noteworthy that energy consumption does not serve as a catalyst for sustained long-term economic growth. Consequently, it becomes imperative to incorporate the principles of energy conservation, emission reduction, and environmental protection into the framework of urban economic development planning. Meanwhile, the effect of energy consumption on variables other than urbanization fluctuated greatly, the impact on environmental quality fluctuated from 0.2 to 0.4, and the impact on economic growth continued to rise to 0.4. The methodology suggested by the study accurately mirrors the actual conditions of the economy and energy use. This helps both the government and businesses understand the trends in economic development and make necessary adjustments to their energy strategies.

## 1. Introduction

Currently, economic and social progress is closely intertwined with the rapid acceleration of urbanization. Statistics reveal that the global urbanization rate (UR) soared from roughly 30% in 1950 to over 55% by 2020, and it is projected to approach nearly 70% by 2050. This urbanization process is inherently linked to the restructuring of national or regional industries, fostering scientific and technological innovation, and advancing social productivity, demonstrating a profound and interconnected relationship between urbanization and economic growth (EG) [1]. On the one hand, urbanization is one of the important manifestations of economic development. Urbanization involves the relative concentration of populations and resources, which promotes economies of scale and agglomeration. Meanwhile, urbanization enriches urban employment opportunities and increases market demand, promoting industrial development and EG. Urbanization also promotes technological innovation and the flow of scientific knowledge. The gathering and exchange of talent further promotes economic development [2]. On the other hand, EG provides impetus and support for urbanization and accelerates the urbanization process, and economic development prompts cities to absorb more labor and capital investment. This brings employment opportunities, attracts a large influx of foreign population, and promotes the urbanization process [3–5]. In the process of urbanization, the industrial structure has undergone a shift from predominantly agricultural to predominantly industrial, and per capita energy consumption (EC) and energy intensity have risen rapidly, making it an important constraint on economic development. In China, for example, between 1980 and 2020, the acceleration of urbanization has led to a sharp increase in EC of nearly five times. Moreover, the rapid growth in EC has not only exacerbated resource shortages and environmental pressures, but also posed a threat to the sustainable development of the economy. Energy is a key factor in promoting EG, and industrial production and urbanization require a large amount of energy support. The stable supply and effective use of energy are directly related to the speed and quality of economic development. Meanwhile, a country’s energy structure directly determines the direction and speed of its economic development. The security and stability of its energy supply are crucial to stable economic development. In addition, since energy is a primary source of greenhouse gas emissions, analyzing EC can help formulate effective emission reduction strategies and policies, promoting the green transformation and sustainable development of the global economy. Currently, China is in a critical period of energy transition, and the optimization of energy structure is of great significance in promoting EG. In 2024, China’s EC intensity decreased by about 2.5%, and EC per unit of GDP continued to decrease [6]. The improvement of energy utilization efficiency has played a positive role in promoting EG. A comprehensive examination of the correlation between energy and economic development is imperative to enhance the efficacy of energy utilization and promote substantial economic advancement. The analysis of the correlation between energy and economic development, EC has become the focus of economic and social development [7].

In this regard, many scholars and policy makers have launched a series of research and analysis. Rehman et al. analyzed the relationship between various types of energy sources and EG in Pakistan based on data from 1975–2019 and studied the dynamic linkages between the variables using linear autoregressive distributed lag technique. Based on data analysis, the government should implement workable policy measures to solve energy and power challenges because energy use, CO_2_ emissions, and GDP per capita have a positive association with economic development [8]. EG, foreign investment, inflation, and population growth all have an impact on sustainable EC. Sadiq et al. used time series data from 1981 to 2019 to assess the correlation between the factors using a time series data model. The results of the analysis were favorable for the formulation of policies related to sustainable EC [9]. Bui et al. investigated the response mechanisms and interrelationships among financial development, EC and EG in the ASEAN region based on a generalized moments panel vector autoregressive (VAR) framework for the period 1981–2021. Empirical analysis findings demonstrated a reciprocal association between financial development and EC, as well as between EG and EC [10]. Using data from 2001 to 2020, Xing et al. used the VAR and decoupling model to examine the static and dynamic links between carbon emissions and economic development in Shanxi Province, China. A foundation for addressing the issue of an excessive reliance on EC in economic development could be found in the data analysis results [11]. Studies by Rehman et al. showed that efficient energy use could directly promote EC. Based on this, Hypothesis 1 (H1) is proposed: There is a two-way Granger causal relationship between EC and GDP, with EC having a positive impact on GDP in the short term. Studies by Sadiq et al. and Bui et al. have shown that GDP growth is usually accompanied by increased EC and environmental pressure. This study infers that this pressure will be directly reflected in environmental quality (EQ) indicators. Therefore, Hypothesis 2 (H2) is proposed: GDP is a Granger cause of EQ, with a negative long-term impact on EQ. Studies by Xing et al. showed a positive causal relationship between GDP growth and EQ. This indicated that carbon emissions could be decoupled from EG through structural adjustments and policy interventions. Based on this, Hypothesis 3 (H3) is proposed: As a country’s economic development stage evolves, its dependence on EC also changes. This change manifests as a non-linear, long-term dynamic relationship between EC and GDP. Meanwhile, EC and energy systems serve as a pivotal nexus, crucially linking the economic and energy frameworks. Therefore, Hypothesis 4 (H4) is proposed: Urban resource utilization is a Granger cause of GDP and EC, and through agglomeration effects, it has a sustained positive impact on both.

In summary, existing research primarily focuses on pairwise relationships. It lacks in-depth analysis of the dynamic interaction mechanisms among the four subsystems of urbanization, economic development, EC, and EQ. Although some studies have focused on the two-way relationship between the economy and the environment, their scope remains limited. Most of these studies overlook the regulatory and intermediary role of urbanization, which serves as a key hub in the transmission paths involving four variables. This oversight makes it challenging to systematically uncover the nonlinear response and feedback mechanisms among various elements, thereby leaving an important theoretical gap in current research. Meanwhile, most of the current enhancements to models concentrate solely on a single aspect, failing to adequately address issues such as heteroscedasticity interference and the efficiency of parameter optimization in high-dimensional economic and energy datasets. This limitation hampers the precision and robustness of the quantitative depiction of long-term dynamic relationships. In addition, when analyzing the long-term dynamic response mode and impact intensity among variables, the existing studies generally lack the analysis and empirical fact support of the key transformation characteristics in the research period. Taking China as an example, from the start of the reform of the urban economic system in 1985 to the in-depth promotion of the double carbon goal in 2022, the UR has jumped from less than 25% to more than 65%, and the per capita EC has increased nearly fivefold, while the EQ has experienced a turning point around 2010 after continuous deterioration – this series of structural changes provide an ideal “natural experiment” scenario for testing the complex dynamic relationship between the four variables. However, the existing research has not made full use of this typical period for system modeling. Therefore, this study aims to make up for the above theoretical and empirical gaps by building a more robust and accurate analysis framework. In view of this, this study selects the VAR model as the core analysis tool, introduces the autoregressive conditional heteroscedasticity (ARCH) model to deal with the heteroscedasticity of data, and uses the differential evolution (DE) algorithm to optimize the parameter estimation, so as to improve the ability of the model to describe complex dynamic relationships. This study selected a representative province of China from 1985 to 2022 as the research object, which completely covered the key stages of the transition from planned economy to market economy, the acceleration of industrialization and the implementation of green development strategy. The selected provinces have both typical industrial base and rapid urbanization process. The evolution track of UR, per capita GDP, EC and EQ comprehensive index is highly consistent with the overall trend of the country, and has strong representativeness and policy reference value.

The contributions of this study are as follows:

(1) It integrates the four subsystems of UR, EG, EC, and EQ into a unified analytical framework, systematically revealing their dynamic interaction mechanisms and filling the theoretical gap in existing research that does not adequately focus on the hub role of UR.
(2) It constructs an improved VAR model that integrates ARCH and DE algorithms, achieving synergistic innovation in heteroscedasticity handling and parameter optimization, thus improving the robustness and accuracy of long-term dynamic relationship characterization.
(3) Based on long-term time-series analysis of typical provinces, it provides empirical evidence of the synergistic evolution of urbanization, EG, and energy and environment, offering more operational theoretical support for the formulation of regionally differentiated policies.

The study is divided into four main sections. The first section describes the economic development and EC issues that society is currently facing and provides an overview of some of the present state of both domestic and foreign research. The second section describes the construction process of the improved VAR model. The third section mainly focuses on experimental analysis, evaluating the performance of the VAR model. The fourth section summarizes the entire study, pointing out the limitations of this study and future prospects.

## 2. Urban economy and ec based on improved VAR modeling

In order to grasp the urban EG and energy use, the study firstly adopts the VAR model to analyze the dynamic connection and influence mechanism between EG and EC, and then introduces the ARCH model and the DE algorithm to optimize the shortcomings of the VAR model.

### 2.1 Design of a VAR-based model for dynamic correlation analysis of urban economy and EC

**Fig 1:**
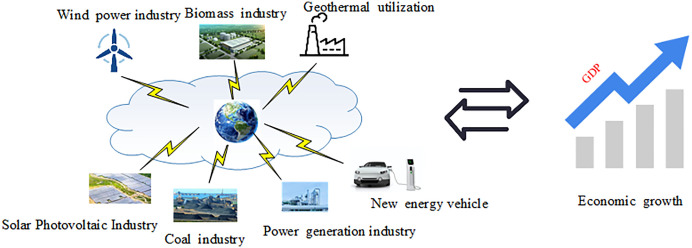
Schematic diagram of the interaction between EG and EC.

Energy is vital for improving productivity, encouraging technological advancement and innovation, speeding up the development of infrastructure, creating jobs, preserving national security, and ensuring energy independence. It also plays a crucial role in economic development [12,13]. However, EG and urbanization exacerbate the increase in EC, and the severity of the EC problem cannot be ignored, and the interaction between the two is shown in Fig 1 [14].

**Fig 2:**
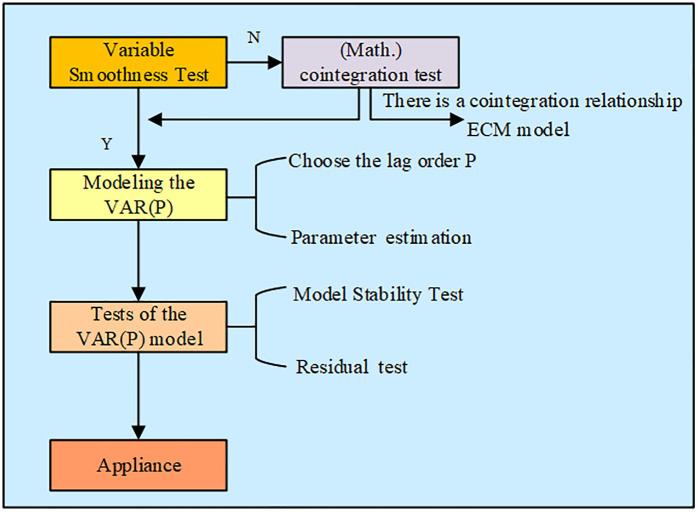
Flowchart of the VAR model construction process.

This study synthesizes previous research in the literature review and constructs an analytical framework centered around four core variables: urbanization, gross domestic product (GDP), EC, and EQ. Based on a literature review, the following research hypotheses are proposed. H1: There is a two-way Granger causal relationship between EC and GDP. This hypothesis stems from the research findings of Rehman et al. [8], which found that energy use has a direct promoting effect on EG, while EG also drives EC to increase in the reverse through scale effects. H2: GDP is a Granger cause of EQ, and its long-term impact is negative. Studies by Sadiq et al. [9] and Bui et al. [10] have shown that GDP growth is usually accompanied by an increase in EC and environmental pressure. Based on this, this study infers that this pressure will be directly reflected in EQ indicators. H3: The dependence of EG on EC will evolve with the stage of economic development, and the two show a dynamic long-term relationship. The research by Xing et al. [11] pointed out that EG and EC can be decoupled through structural adjustment and policy intervention, indicating that the relationship between the two is not constant, but has time-varying characteristics. This study will characterize the evolution trajectory of this dynamic relationship through the impulse response function of the VAR model. H4: Urbanization is a Granger cause of EG and EC, and exerts a sustained driving effect on both. Based on this, this study selects four factors, UR, GDP, EC, and EQ, for quantitative analysis. This analysis aims to comprehensively examine energy issues in the context of economic and social development and deeply analyze the relationship between EQ and carbon emissions. The UR dataset uses raw data from statistical yearbooks. GDP is calculated at constant 2000 prices and adjusted for inflation. The EC dataset summarizes consumption of various energy sources, including coal, oil, natural gas, and electricity, converting them to a standard coal equivalent. The EQ dataset is constructed using principal component analysis and four negative indicators are selected: sulfur dioxide emissions, industrial wastewater emissions, industrial solid waste generation, and annual average PM2.5 concentration. First, these indicators are standardized using Z-scores. Then, a principal component is extracted based on an eigenvalue greater than one. The overall score is calculated using the variance contribution rate of each indicator as a weight. A higher score indicates worse EQ. Outliers are identified using the 3σ principle and corrected using linear interpolation. To reduce heteroscedasticity, all variables undergo natural logarithmic transformation, after which ADF tests are performed on the transformed series. All raw data has been organized and uploaded to the Dryad digital repository under a CC0 1.0 Public Domain Donation License. Conventional variable association analysis is predicated on economic theory for qualitative description. However, the economic theory analysis principally focuses on the causal relationship between the variables, and it is incapable of providing specific numerical explanations, which makes it difficult to explain the severity of the association. The VAR model has the capacity to elucidate the dynamic relationship between two or more variables over a sample period by means of vector autoregression, thereby reflecting the long-term and dynamic relationship between variables. Moreover, the VAR model focuses on the relationship between two or more variables, reducing the need for data volume. In comparison with conventional regression methodologies, VAR models circumvent the challenges associated with regression equation selection and mitigate the computational intricacy inherent in addressing multiple regressions. On the other hand, a statistical model called the VAR model is employed to examine the connection between several linked time series variables [15]. Therefore, in order to solve the problems of theoretical analysis, the study selected the VAR model for variable analysis. To help with the direction of economic activities and policy making, historical data on the four effects of urbanization, economic development, EQ, and EC are analyzed in order to predict the development trend of future variables [16,17]. VAR is an extension of autoregressive model, applicable to the situation where there are mutual influence and linkage between multiple variables, the construction process of VAR model is shown in Fig 2.

The VAR model operates under the assumption that the relationship between multiple variables can be explained by their own past values and the past values of each other. The variable relationships are handled in an endogenous variable treatment, which reduces the uncertainty caused by subjective judgment errors to some extent. Equation (1) defines the VAR model’s expression.


$$R_{t}=\theta +\omega_{ij}\sum_{i=1}^{P}R_{i,t-n}+\delta_{t}$$ (1)

In Equation (1), $R_{t}=\left(R_{1,t},R_{2,t},R_{3,t},R_{4,t}\right)$ is a four-dimensional vector of the developmental states of the four variables, urbanization, economic development, EC and EQ, at moment t. θ is the constant matrix with length of the variables and $\omega_{ij}$ denotes the coefficient matrix. P is the endogenous variables. n denotes the lag term and $\delta_{t}$ denotes the random perturbation term. The prediction of future observations is calculated by the parameter estimation method of the VAR model and the related statistical analysis is performed.

There are some difficulties and shortcomings in the VAR model in analyzing urban economy and EC analysis. On the one hand, the data related to urban economic development and energy utilization involve different industries and sectors, and have a dynamic trend of continuous change. However, the establishment of VAR model is usually based on the overall data, ignoring the differences between the data, and the model is less capable of capturing the characteristics and relationships at different scales. On the other hand, VAR models rely on adequate, continuous and high-quality time-series data, and restricted data sources and missing data may lead to serious deviations between model calculations and actual results [18].

To accurately capture the short-term changes of variable data and reduce the computational error, the study introduces ARCH together with VAR model to construct the effect analysis model between urban economy and EC. ARCH is an econometric model that describes the heteroscedasticity in time series data, which can solve the untenable problem that the variance of the error term of the traditional linear regression model is a constant [19]. The ARCH model used in the study is generalized autoregressive conditional heteroscedasticity model (GARCH). The GARCH model, a development of the ARCH model, incorporates a past conditional heteroscedasticity term, accounting for the past squared error term and the impact of past variance on current variance. This model’s capacity to address long-term dependence and nonlinear time series patterns enhances its adaptability to modeling requirements.

The GARCH (n,m) model is selected for the study to analyze the variable effects, n and m denote the lag order of the ARCH and GARCH parts, respectively. Moreover, the expression of the sequence of observations is shown in Equation (2).


$$\varepsilon_{t}=\sigma_{t}*{\tilde{e}}_{t}$$ (2)

In Equation (2), $\varepsilon_{t}$ denotes the sequence of observations. $\sigma_{t}$ denotes the heteroskedasticity term, which satisfies the GARCH (n,m) model. ${\tilde{e}}_{t}$ denotes the white noise sequence that satisfies the independent homogeneous distribution.

The expression for the GARCH (n,m) model is shown in Equation (3).


$${\sigma_{t}}^{\text{2}}=\alpha_{\text{0}}+\alpha_{\text{1}}\overset{\text{2}}{\underset{t-1}{\varepsilon }}+...+\alpha_{n}\overset{\text{2}}{\underset{t-n}{\varepsilon }}+\beta_{\text{1}}\overset{\text{2}}{\underset{t-1}{\sigma }}+...+\beta_{m}\overset{\text{2}}{\underset{t-m}{\sigma }}$$ (3)

In Equation (3), $\alpha_{\text{0}}$ denotes a non-negative constant. $\alpha_{\text{1}}$,..., $\alpha_{n}$ denote non-negative coefficients. $\beta_{\text{1}}$, $\beta_{\text{2}}$... $\beta_{m}$ denote non-negative coefficients. The study uses the augmented Dickey-Fuller test (ADF) to identify whether or not the data need to be smoothed before constructing the model in order to prevent the pseudo-regression problem produced by unsteady data. The GARCH-VAR model is often created based on smooth time series data.

**Fig 3:**
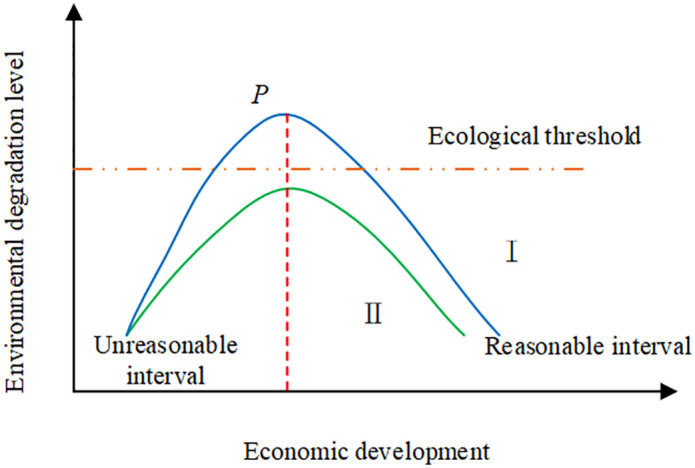
Schematic diagram of the EKC for GDP and EQ.

The environmental Kuznets curve (KC) is also used in the study to examine the connection between EQ and economic development. Fig 3 displays the environmental KC, sometimes referred to as the “U-shaped curve”. The environmental KC in Fig 3 is separated into three parts: the highest point, the reasonable interval, and the unreasonable interval. The unreasonable interval represents the early stage of economic development, the process of industrialization and urbanization, and the EG is often accompanied by the increase of EC and environmental pollution. A reasonable interval is indicative of an increase in income level, social awareness of sustainable development, the gradual enhancement of environmental protection awareness, measures taken by the state, technological progress, and optimization of economic structure for the purpose of controlling environmental problems. The highest point represents the peak of environmental degradation.

### 2.2 Design of GARCH-VAR model incorporating improved DE algorithm

**Fig 4:**
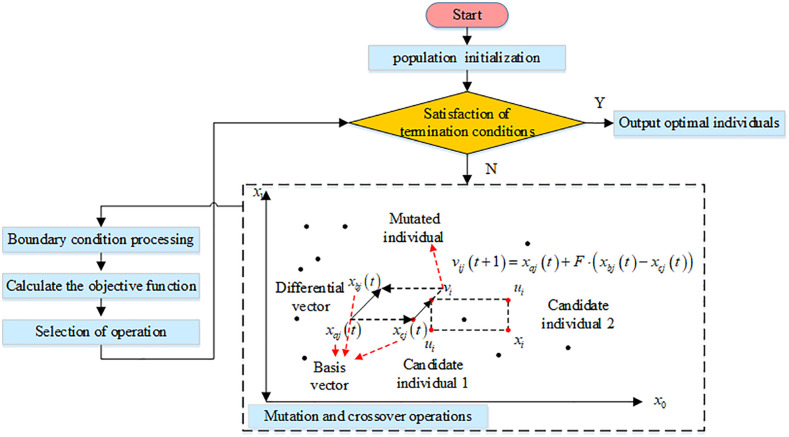
Differential evolutionary algorithm working mechanism and flow.

The improved VAR model involves more parameter estimation and variable selection, which affects the solution optimization, prediction performance and model interpretability. To reduce the prediction error of the model, this experiment introduces the DE algorithm in intelligent optimization algorithms for parameter and variable optimization. DE is an evolutionary algorithm based on the differences between individuals of a group, which simulates the cooperation and competition process of individuals between groups [20]. The DE algorithm works on a straightforward, easily implementable premise with fewer control parameters. The DE method uses an iterative procedure to consecutively carry out mutation, crossover, and selection operations to pick the genes of the optimal individuals as the problem solution [21]. In this process, the genes of individuals represent the possible solutions of the problem to be solved. The DE method, whose working mechanism is depicted in Figure 4, has superior overall performance and is frequently used to identify the best answer to a variety of issues.

The feasible solution space of the DE algorithm is set as $\Omega =\overset{D}{\underset{j=1}{\prod }}\left[\overset{low}{\underset{j}{x}},\overset{up}{\underset{j}{x}}\right]$. $\overset{low}{\underset{j}{x}},\overset{up}{\underset{j}{x}}$ denotes the upper and lower boundary conditions of the population individuals. It generates a random beginning population of a predetermined number of people and sets the size of that population based on the optimization requirement. Equation (4) provides the expression’s definition.


$$x_{ij}\left(0\right)=\overset{low}{\underset{j}{x}}+rand\left[0,1\right]\cdot \left(\overset{up}{\underset{j}{x}}-\overset{low}{\underset{j}{x}}\right)$$ (4)

In Equation (4), the i th individual of the population is defined as $x_{i}\left(t\right)=\left\{x_{i1}\left(t\right),x_{i2}\left(t\right),...,x_{ij}\left(t\right),...,x_{iD}\left(t\right)\right\}$, where j denotes the variable and j={1,2,...,D}, D denote the dimensions. t is the current iterations.

The population dominant individual is calculated according to the fitness function and the mutation operation in the individual is performed. The gene of one more individual is selected as the base and utilize the difference of different individuals to constitute the differential gene. Equation (5) illustrates the computation process of creating a new individual by adding the genes of the base individual and the differential genes.


$$v_{ij}\left(t+1\right)=x_{aj}\left(t\right)+F\cdot \left(x_{bj}\left(t\right)-x_{cj}\left(t\right)\right)$$ (5)

In Equation (5), $v_{ij}\left(t\right)$ denotes the variant individual, and $x_{aj}\left(t\right)$, $x_{bj}\left(t\right)$, and $x_{cj}\left(t\right)$ denote three different base individuals. F denotes the mutation operator. The new individuals are crossed over with the parent-based individuals as part of the evolutionary process to promote population diversity. Equation (6) depicts the operation procedure.


$$u_{ij}\left(t+1\right)=\left\{ \begin{array}{c} @l@v_{ij}\left(t+1\right)\;\;if\;rand\left(0,1\right)\leq C_{R}\;or\;j=j_{rand}\\ x_{ij}\left(t\right)\;\;if\;rand\left(0,1\right)>C_{R}\;or\;j=j_{rand} \end{array}\right.$$ (6)

In Equation (6), $u_{ij}\left(t+1\right)$ denotes the variance variable and $C_{R}$ denotes the crossover operator. $j_{rand}$ denotes the random dimension. Finally, the selection operation is performed according to the greedy criterion to compare the new individual with the corresponding individual of the parent. Equation (7) illustrates the procedure of calculation.


$$x_{i}\left(t+1\right)=\left\{ \begin{array}{c} @l@u_{i}\left(t+1\right)\;\;if\;f\left(u_{i}\left(t+1\right)\right)\leq f\left(x_{i}\left(t\right)\right)\\ x_{i}\left(t\right)\;\;if\;f\left(u_{i}\left(t+1\right)\right)>f\left(x_{i}\left(t\right)\right) \end{array}\right.$$ (7)

In Equation (7), f denotes the fitness function. Although the DE algorithm helps to improve the parameter estimation and variable selection of the VAR model. However, the DE algorithm may face high-dimensional optimization problems when applied to VAR models. In the process of calculating the VAR model, it is imperative to note that the variables and lag orders have the effect of increasing the number of parameters. Conventional DE algorithm is susceptible to convergent local optimum, diminished computational efficiency, and slipping into a local optimum [22].

The study suggests a DE algorithm that incorporates cluster analysis. To finish the population optimization at the early stage of the algorithm, representative individuals are extracted from the original population using cluster analysis, and poorer individuals are replaced. Secondly, on the basis of clustering, adaptive strategies are designed to improve the variation and crossover strategies of DE. The clustering method employed in the present study is K-modes clustering, which is an extension of the k-means algorithm. K-modes clustering is primarily used for the clustering of discrete data and is suitable for the division of populations. All raw data used in this study are obtained from publicly available sources. The processed dataset has been uploaded to the Dryad digital repository and is licensed under the CC0 1.0 Public Domain Donation License, making it freely available without authorization. The Python code used for model building, algorithm optimization, and results analysis is open source on GitHub and includes detailed comments and instructions. The set of clustered samples is defined as $X=\{x_{\text{1}},x_{\text{2}},...,x_{n}\}$. The value of the attribute $\{A_{\text{1}},A_{\text{2}},...,A_{m}\}$ of the sample point $x_{i}=\{x_{i1},x_{i2},...,x_{im}\}$ is calculated in Equation (8).


$$Dom\left(A_{i}\right)=\{\overset{i}{\underset{\text{1}}{a}},\overset{i}{\underset{\text{2}}{a}},...,\overset{i}{\underset{l}{a}}\}\;$$ (8)

The K-modes clustering method uses the Hamming distance measure to calculate the similarity between populations. Equation (9) displays the computation method.


$$D\left(x_{i},x_{j}\right)=\overset{m}{\underset{l=1}{\sum }}d\left(x_{il},x_{jl}\right)\;d\left(x_{il},x_{jl}\right)=\left\{ \begin{array}{c} @c@0\;x_{il}=x_{jl}\;\\ 1\;x_{il}\neq x_{jl}\; \end{array}\right.$$ (9)

In Equation (9), $x_{il}$ and $x_{jl}$ denote two arbitrarily different populations. In the process of selecting the clustering center, the study introduces the minimization of sum of squared error (SSE) to calculate the SSE value of all individuals, and the minimum value individual is determined as the initial clustering center of the clusters. Additionally, Equation (10) illustrates the computation procedure.


$$SSE=\sum_{l=1}^{k}\sum_{X\in L_{l}}Dist{\left(x,Z_{l}\right)}^{\text{2}}$$ (10)

**Fig 5:**
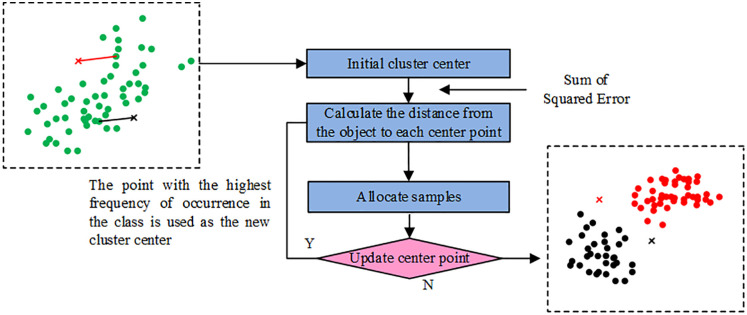
Schematic diagram of the improved K-modes clustering process.

In Equation (10), k is the clusters and $Z_{l}$ is the clustering center of the l th cluster $L_{l}$. $Dist\left(x,Z_{l}\right)$ denotes the similarity between the sample x and the clustering center. In summary, the research improved K-modes clustering process is shown in Figure 5.

As shown in Fig 5, K-modes clustering introduces SSE method to determine the initial cluster center, on the basis of which it continues to complete the division of data samples. After completing the initial population delineation using the improved K-modes clustering, the study introduces an adaptive strategy in the mutation and crossover process. The mutation operator F is used to generate new individuals by performing mutation operations on the parent individuals. The DE method converges slowly and has a tendency to search worldwide when the mutation operator F is big. The DE method prefers to search locally when the mutation operator F is small, and it is simple to converge, but it is also simple to enter the local optimum. The adaptive mutation operator adjustment process is shown in Equation (11).


$$F=F_{max}-\left(F_{max}-F_{min}\right){\left(\frac{t}{T}\right)}^{\text{2}}$$ (11)

In Equation (11), $F_{max}$ and $F_{max}$ denote the scaled maximum and minimum values of the mutation operator. t, T denote the current, maximum iteration number, respectively. The crossover operator $C_{R}$ is the parameter that controls the crossover probability. The adaptive crossover strategy designed by the study is shown in Equation (12).


$$CR_{t}=\left\{ \begin{array}{c} @l@\frac{1+cost}{\text{2}}\;\;mod\left(t,p\right)=0\\ CR_{t-1}\;\;otherwise \end{array}\right.$$ (12)

In Equation (12), mod(t,p) denotes one update per iteration p times.

The formula for determining the aggregation of DE algorithm populations is shown in Equation (13).


$$C=\sum_{j=1}^{D}\sum_{i=1}^{NP}\left|x_{i,j}-\overset{―}{x_{j}}\right|$$ (13)

In Equation (13), NP denotes the population size, $\overset{―}{x_{j}}$ denotes the individual vector mean of the population in the j th dimension, and C denotes the population aggregation degree. The study presents the perturbation dimension variation technique to lead the population towards the global optimum, preventing individuals within the population from falling into the local optimum. The calculation process of the perturbation dimension variation strategy is shown in Equation (14).


$$\overset{t}{\underset{i,j}{x}}=C⬝\overset{t}{\underset{best,j}{x}}+\left(1-C\right)\overset{t}{\underset{r1,j}{x}}+\alpha \left(\overset{t}{\underset{r2,j}{x}}-\overset{t}{\underset{r3,j}{x}}\right)$$ (14)

**Fig 6:**
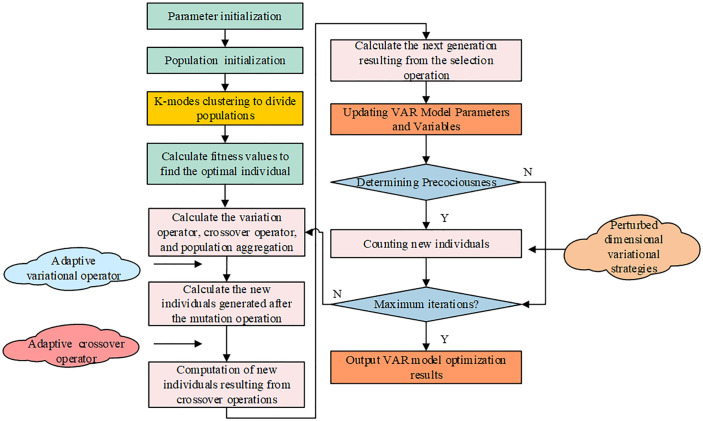
Improved DE algorithm to optimize VAR model flowchart.

In Equation (14), α denotes the accelerated random perturbation coefficient. In summary, the improvement and optimization of DE algorithm is completed by combining all the improvement measures, and the operation flow is shown in Fig 6. The improved DE algorithm introduces adaptive variation operator, crossover operator and perturbation dimension variation strategy after K-modes clustering to divide the population. The enhanced DE technique is utilized to enhance the VAR model’s parameter estimation and variable optimization, ultimately concluding the dynamic analysis between EC and urban economic development.

## 3. Performance testing of models and correlation analysis of economic and energy dynamics

To verify the performance of the VAR model designed by the study and its application, the study designs a performance test of the improved algorithm and a dynamic correlation analysis of the variables. The results of this analysis are discussed in the following section.

### 3.1 Improved performance testing for DE

Hardware environment used for the experiment The operating system is Windows 10, the processor is AMD Ryzen 7 5800H with Radeon Graphics 3.20 GHz, the graphics processor is NVIDIA GeForce RTX 3070 (Laptop, 130W), and the RAM is 64 G. The software environment Python language development environment is pycharm. Set the dimension D = 50 and the population size NP is 50. The crossover operator is adjusted every 50 iterations, with the variation operator scaling value having maximum and minimum values of 0.9 and 0.2, respectively. The CEC2022 test function set is chosen for validation. CEC2022 contains 12 benchmark functions covering single-peak, multi-peak, hybrid and composite functions. F1 in the CEC2022 function set is a single-peak function, F2-F5 are multi-peak functions, F6-F8 are hybrid functions, and F9-F12 are composite and multimodal functions. In a comparative experiment, the upgraded DE algorithm is compared with the traditional DE algorithm and the traditional genetic algorithm (GA) to validate the performance of the research design algorithm with respect to evolutionary trends and parameter optimization.

**Fig 7:**
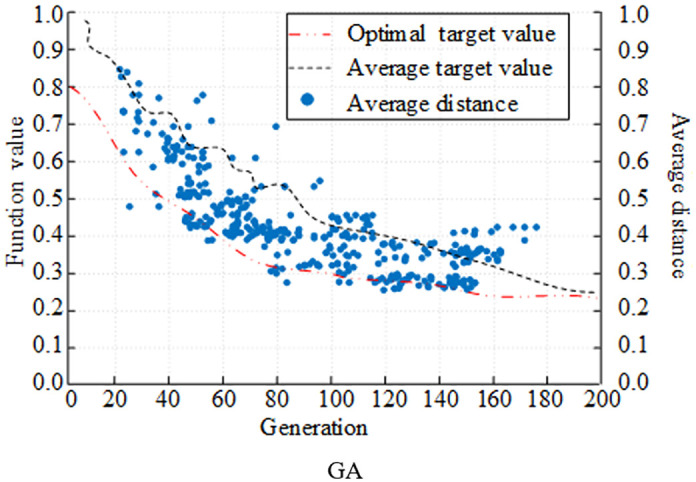
Comparison of population evolution of different algorithms (GA).

**Fig 8:**
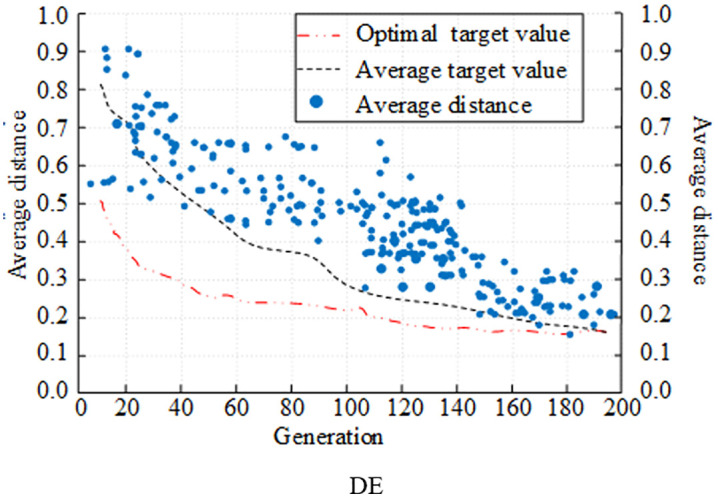
Comparison of population evolution of different algorithms (DE).

**Fig 9:**
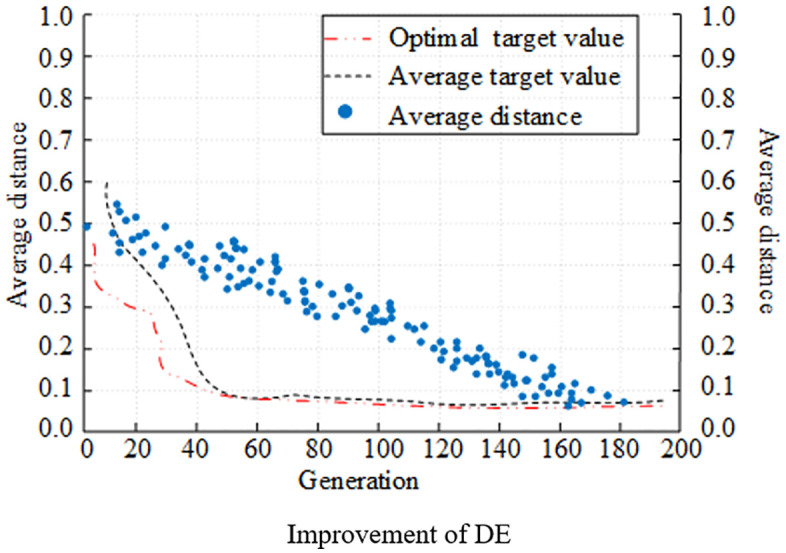
Comparison of population evolution of different algorithms (Improvement of DE).

First, the population fitness curve is utilized as the assessment index, and the evolutionary capabilities of various algorithms are compared and examined. Fig 7 displays the experimental results. In Fig 7, the traditional GA is faster in the pre-evolutionary stage, the evolutionary speed slows down around 100 generations, and only gradually approaches the optimal population fitness in 180 generations. In Figs 8 and 9, the convergence target value of the population fitness curve of DE algorithm was lower than 0.2, which was smaller than that of GA. A comparison of the DE algorithm in its original state and in its improved state revealed that the algorithm with the improved strategy approached the optimal population fitness in the early stage of evolution in approximately 40 generations. The clustering division of the population with the adaptive strategy improved the population fitness ability. In the meantime, it is evident from comparing the average distance curves of the three algorithms that the population of the improved DE algorithm maintained a better average distance during the evolution process, and that the distance gradually decreased as the algorithm evolved.

**Fig 10:**
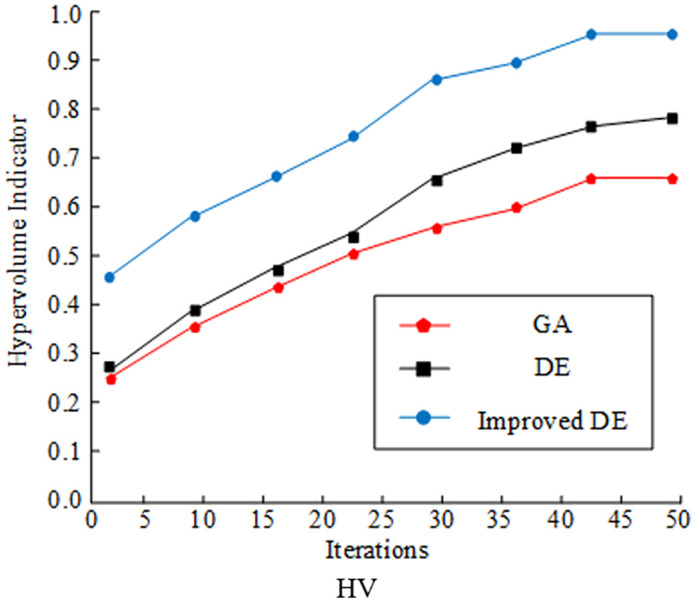
Comparison of HV and IGD for different optimization algorithms (HV).

**Fig 11:**
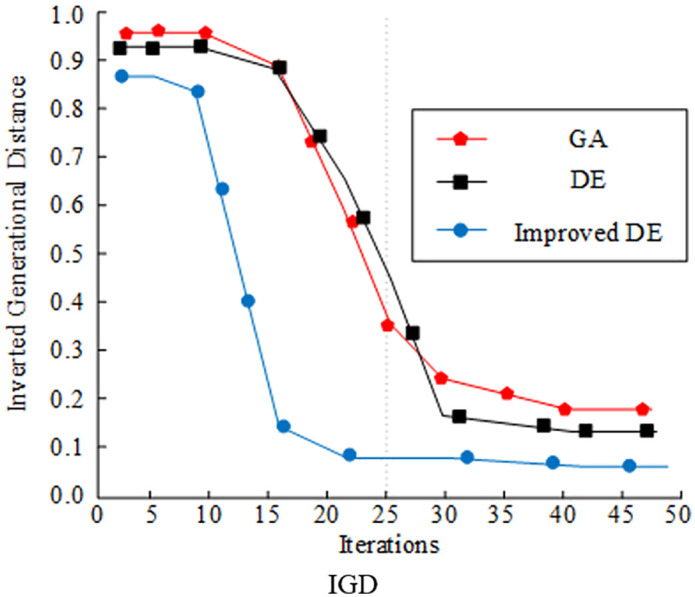
Comparison of HV and IGD for different optimization algorithms (IGD).

Hypervolume indicator (HV) and inverted generational distance (IGD) are selected as the evaluation indices for comparing and analyzing the optimization seeking ability of various algorithms. The experimental results are displayed in Fig 8. In Fig 10, the improved DE algorithm had the largest HV value under the optimization of the improved strategy, and the maximum HV value reached 0.92. In contrast, the HV value of the traditional GA and DE algorithms only reached the level of 0.65 and 0.79. The IGD values of several algorithms are shown in Fig 11 decreasing as the number of iterations increased. The IGD value of the improved DE converged at 0.08, which was lower than the GA and DE algorithms by 0.19 and 0.08, respectively. Comprehensively, the algorithms designed by the study had better convergence performance and distributional performance in solving.

### 3.2 Effects of the application of improved VAR models

In consideration of China’s economic transition from a planned economy to a market economy since 1985, accompanied by a series of pivotal economic policies and social transformations, the economic, social, and environmental shifts that have ensued are indicative of the interplay between UR, EG, EC, and EQ. Therefore, the 1985–2022 time dimension is used to collect economic and energy-related data for a province in China, including gross regional product, total consumption of various energy sources, urban and township resident population, and the amount of various pollutants generated. Principal component analysis is employed to facilitate the downscaling and comprehensive processing of the data. The four variables of urbanization, economic development, EC, and EQ are denoted as UR, GDP, EC, and EQ, respectively. UR is the ratio of urban population to total population. GDP is the ratio of gross regional product to total population. EC is the ratio of various end-use EC to total population. Moreover, EQ is the indicator obtained by analyzing sulfur dioxide, air pollutants, water pollutants, and solid pollutants. The data are sourced from the official website of the National Bureau of Statistics of China (http://www.stats.gov.cn), its statistical yearbooks, and the economic, environmental, and population statistics published by the provincial statistical bureaus (specifically those of the provinces selected for the study). Additional data come from local government websites, the Ministry of Ecology and Environment, local environmental protection departments, and the National Energy Administration and provincial energy management departments. The raw data are recorded in Excel/CSV format with a continuous time series and no missing data. Outliers caused by statistical method adjustments are corrected using linear interpolation. All data acquisition follows the data disclosure standards of relevant departments. Principal component analysis is used to reduce the dimensionality of the data and construct comprehensive indicators, and STATA 17.0 is used for subsequent statistical analysis.

First, a smoothness test is performed using the ADF test, a unit root test, with the null hypothesis that the series has a unit root (non-stationary). The test results are shown in Table 1. Among them, dln represents the first-order differencing form of the variables. In Table 1, the ADF statistic for all variables after first differencing was less than the 1% confidence level critical value, and the corresponding *p*-values were all less than 0.01. Therefore, the null hypothesis was rejected, indicating that all variables reached a stationary state after first differencing. This satisfied the basic requirement of a VAR model, namely that the data must exhibit stationarity.

**Table 1:** Sequential smoothness test results.

Variant	ADF statistic	1% threshold	5% threshold	10% threshold	*p*	Conclude
dlnUR	−4.12	−3.78	−2.96	−2.61	0.003	Steady
dlnEC	−4.36	−3.78	−2.96	−2.61	0.002	Steady
dlnGDP	−3.94	−3.78	−2.96	−2.61	0.05	Steady
dlnEQ	−6.19	−3.78	−2.96	−2.61	0.00	Steady

After completing the ADF test, lag order estimation was performed using five criteria: likelihood ratio test, final prediction error, Akaike information criterion, Hannan-Kunz information criterion, and Schwarz information criterion. The results are shown in Table 2. In the likelihood ratio test, the chi-square statistic for lag 4 was 39.44, with a *p*-value of 0.00. Since this was less than 0.05, the null hypothesis was rejected. This result was also better than lags 1, 2, and 3. The Akaike information criterion value for lag 4 was −7.48, which was slightly higher than the AIC value of −7.74 for lag 1. However, it was significantly better than the AIC values of −7.38 for lag 2 and −7.21 for lag 3. The final prediction error for lag 4 was 1.5e-08, significantly lower than the final prediction errors of other lag orders. The Hannan-Kunz criterion value for lag 4 was −6.51, only slightly higher than lag 3. The Schwarz criterion value for lag 4 was −4.24, which was better than other lag orders. Lag 1 was missing key dynamic information. Lag 2 did not have a significant likelihood ratio test. Lag 3 showed limited improvement in fit. Therefore, the final lag order was determined to be 4.

**Table 2:** Estimated results of lag order of ADF test.

Lag	LL	Likelihood ratio test	df	P	Final Prediction Error	Akaike Information Guidelines
0	106.46	/	/	/	5.3e-09	−7.64
1	124.16	34.19	16	0.00	4.8e-09	−7.74
2	135.36	20.09	16	0.20	8.2e-09	−7.38
3	148.24	27.49	16	0.04	1.2e-08	−7.21
4	167.49	39.44	16	0.00	1.5e-08	−7.48
**Lag**	**LL**	**Likelihood ratio test**	**df**	**P**	**Hannan-Kunz Guidelines**	**Schwarz Information Guidelines**
0	106.46	/	/	/	−7.64	−7.48
1	124.16	34.19	16	0.00	−7.50	−6.81
2	135.36	20.09	16	0.20	−6.84	−5.47
3	148.24	27.49	16	0.04	−6.47	−4.71
4	167.49	39.44	16	0.00	−6.51	−4.24

Before establishing a VAR model, it was necessary to test the long-term equilibrium relationship among the variables. This study used the Johansen cointegration test to examine whether there was a cointegration relationship among the four variables UR, GDP, EC, and EQ. The results are shown in Table 3. Both the trace test and the maximum eigenvalue test rejected the null hypothesis that there was no cointegration relationship (r = 0) at the 5% significance level, and at least three cointegrating vectors existed among the four variables. This indicated that there was a long-term stable equilibrium relationship among these four variables, satisfying the basic conditions for establishing a VAR model.

**Table 3:** Johansen cointegration test results.

Null hypothesis	Eigenvalues	Trace statistic	5% critical value	*p* value	Maximum eigenvalue statistic	5% critical value	*p* value
r = 0	0.682	98.47	47.21	0.000	46.23	27.07	0.000
r ≤ 1	0.514	52.24	47.21	0.000	29.86	20.97	0.002
r ≤ 2	0.387	22.38	15.41	0.004	18.42	14.07	0.011
r ≤ 3	0.102	3.96	3.76	0.047	3.96	3.76	0.047

*Note: r represents the number of cointegrating vectors, and the test includes the intercept and trend terms.*

This study verified the stability of the VAR model by calculating the reciprocal moduli of the model’s eigenvalues. Table 4 shows the values of the reciprocal moduli of all eigenvalues, with a maximum value of 0.946 and a minimum value of 0.423. All the reciprocal moduli of the eigenvalues were less than 1. This indicated that the established VAR model met the stability requirements and could be used for subsequent impulse response analysis and variance decomposition.

**Table 4:** Stability test results of the VAR model.

Characteristic root number	Real part of characteristic root	Imaginary part of characteristic root	Modulus of characteristic root
1	0.892	0.156	0.906
2	0.892	−0.156	0.906
3	0.843	0.287	0.891
4	0.843	−0.287	0.891
5	0.768	0.412	0.872
6	0.768	−0.412	0.872
7	0.723	0.385	0.819
8	0.723	−0.385	0.819
9	0.654	0.267	0.706
10	0.654	−0.267	0.706
11	0.546	0.183	0.576
12	0.546	−0.183	0.576
13	0.512	0.000	0.512
14	0.445	0.000	0.445
15	0.423	0.000	0.423
16	0.389	0.000	0.389

*Note: The model contains 4 endogenous variables and a total of 16 eigenvalues.*

This study used the Portmanteau Q test and the LM test to examine the autocorrelation of the VAR model residuals. The results are shown in Table 5. The Portmanteau Q statistic was not significant at any lag order, with *p*-values greater than 0.05. The LM test also accepted the null hypothesis that the residuals were not autocorrelated. This indicates that there is no autocorrelation problem in the model residuals, and the model specification is reasonable.

**Table 5:** Results of residual autocorrelation test.

Test method	Lag order	Statistic	P value
Portmanteau Q	4	28.47	0.162
Portmanteau Q	8	42.13	0.214
Portmanteau Q	12	56.89	0.298
LM test	4	18.24	0.196
LM test	8	22.57	0.308

Economic and energy-related time series data often exhibit fluctuations and clustering. This study conducted an ARCH effect test on the residuals of the VAR model. Table 6 lists the LM test results for the squared residual series. A significant ARCH effect was observed under lags from first to fourth order, with *p*-values less than 0.05. The residual series exhibited conditional heteroscedasticity, which also demonstrated the reasonable basis and practical necessity for introducing ARCH or GARCH models to optimize the original VAR model in this study.

**Table 6:** Results of ARCH effect test.

Lag order	LM statistic	P value
1	8.56	0.003
2	12.34	0.002
3	14.78	0.001
4	15.92	0.000

This study used the bootstrap method to calculate confidence intervals for impulse response functions to improve the reliability of the analysis results. Setting the bootstrap method to 2000 repeated samplings allows for the calculation of 95% confidence intervals for the impulse responses of all variables. Table 7 shows the estimated impulse response points and upper and lower bounds of the bootstrap confidence intervals for the main variable combinations from period 1 to period 10. Table 7 shows that EC had a significant negative effect on EQ in the early stages, with no zero values appearing in the corresponding confidence intervals. This state continued until period 6, after which the confidence intervals began to include zero. The two had a significant short-term effect, but the long-term correlation was no longer significant. GDP’s effect on EQ exhibited similar characteristics. The negative impact was prominent in the early stages, but the correlation gradually weakened and became insignificant in the later stages. In the process of UR affecting GDP, the confidence intervals for each period do not contain zero. The correlation response value increased from 0.042 in period 1 to 0.387 in period 10, indicating that urbanization had a sustained and significant positive driving effect on EG. GDP had a significantly positive correlation with EC in the early stages, and the confidence interval did not reach 0. The response value gradually decreased from 0.184 in period 2 to 0.072 in period 10. Simultaneously, the range covered by the confidence interval also continuously narrowed. This data supports hypothesis H3, which states that the effect of EG on EC exhibits a non-linear, gradually weakening characteristic. UR’s impulse response to EC also shows a consistently significant positive impact. The response value increased from 0.031 in period 1 to 0.174 in period 10. The results of the actual analysis also directly confirm hypothesis H4, indicating that urbanization development has a driving effect on EC throughout its entire process.

**Table 7:** Impulse response analysis results based on bootstrap method.

Response combination	Lag period	Response value	Lower limit of 95% confidence interval	Upper limit of 95% confidence interval
EC → EQ	1	−0.087	−0.124	−0.052
	2	−0.103	−0.148	−0.061
	4	−0.076	−0.115	−0.038
	6	−0.041	−0.089	0.006
	8	−0.023	−0.068	0.021
	10	−0.015	−0.057	0.026
UR → GDP	1	−0.065	−0.102	−0.029
	2	−0.091	−0.134	−0.049
	4	−0.058	−0.097	−0.021
	6	−0.032	−0.078	0.013
	8	−0.018	−0.063	0.026
	10	−0.011	−0.054	0.031
GDP → EQ	1	0.042	0.018	0.067
	2	0.087	0.054	0.119
	4	0.152	0.108	0.196
	6	0.221	0.167	0.275
	8	0.294	0.229	0.359
	10	0.387	0.311	0.463
GDP → EC	1	0.156	0.112	0.199
	2	0.184	0.136	0.232
	4	0.152	0.108	0.195
	6	0.117	0.069	0.165
	8	0.091	0.043	0.139
	10	0.072	0.024	0.120
UR → EC	1	0.031	0.011	0.052
	2	0.031	0.053	0.086
	4	0.089	0.053	0.126
	6	0.119	0.074	0.164
	8	0.147	0.092	0.202
	10	0.174	0.108	0.240

The improved VAR model was established after the ADF test, and the lag order was set to 4. The Granger causality test of the improved VAR model was carried out first, and the test results are shown in Table 3. In Table 8, “Chi^2^” denotes the chi-square statistic and “df” denotes the degree of freedom. As shown in Table 8, GDP, EC, and the two jointly UR are Granger causes of EQ, where economic development and EC jointly catalyze the deterioration of EQ, with significance levels lower than 0.1 and 0.05, respectively. The process of urbanization is a Granger cause of economic development, with significance levels lower than 0.1. EQ, GDP, and the two jointly UR are Granger causes of EC, and EQ, EC, and the two jointly GDP are Granger causes of UR. It is evident that the impacts of economic development and EC are not monolithic and unidirectional. The promotion of EG and the regulation of EC should be contemplated in conjunction with the urbanization process and EQ.

**Table 8:** VAR model granger test results.

Equation	Excluded	Chi^2^	df	P value
EQ	GDP	7.91	4	0.06
EQ	EC	10.89	4	0.02
EQ	UR	2.13	4	0.97
EQ	ALL	27.16	12	0.01
GDP	EQ	7.06	4	0.21
GDP	EC	3.54	4	0.64
GDP	dlnUR	8.49	4	0.07
GDP	ALL	19.06	12	0.08
EC	EQ	16.19	4	0.00
EC	GDP	15.16	4	0.01
EC	dlnUR	6.94	4	0.23
EC	ALL	39.19	12	0.00
UR	EQ	20.76	4	0.00
UR	GDP	6.21	4	0.27
UR	EC	7.09	4	0.09
UR	ALL	32.06	12	0.00

To further test Hypothesis H3, a recursive estimation method was used to examine the changing trend of the impulse response coefficient of GDP to EC across different time windows, to identify the time-varying characteristics of their relationship. Table 9 shows the impulse response coefficient of GDP to EC and its trend with time windows under different lag periods. Looking at the entire sample period, the impulse response of GDP to EC initially showed a positive impact, reaching a peak of 0.184 in period 2 before gradually weakening, stabilizing at around 0.072 by period 10. This indicated that EG had a strong pulling effect on EC in the short term, but its long-term impact tended to weaken. Looking at different time periods, the peak value of the impulse response of GDP to EC was higher during 1985–2000, reaching 0.216, and the decay rate was slower. However, during 2001–2022, the peak value decreased significantly and the decay rate accelerated. This trend verifies Hypothesis H3, namely, that as the economic development stage transitions from the early stage of industrialization to the stage of high-quality development, the dependence of EG on EC exhibits a non-linear decreasing characteristic. Policy intervention, technological progress, and industrial structure upgrading are key factors driving the evolution of this relationship.

**Table 9:** Time-varying characteristics of GDP impulse response coefficient to EC.

Lag period	Full sample period (1985–2022)	Time period I (1985–2000)	Time period II (2001–2022)
1	0.156	0.182	0.128
2	0.184	0.216	0.142
3	0.171	0.203	0.135
4	0.152	0.187	0.121
5	0.133	0.169	0.108
6	0.117	0.152	0.096
7	0.103	0.152	0.085
8	0.091	0.124	0.076
9	0.081	0.113	0.076
10	0.072	0.105	0.062

To further test Hypothesis H4, cumulative impulse response analysis was used to quantify the long-term impact of UR on GDP and EG, thus verifying the persistence of the agglomeration effect. Table 10 shows the cumulative impulse response results of UR to GDP and EC. The cumulative response coefficient of UR to GDP increased from 0.152 in period 4 to 0.387 in period 10, showing a continuous increase with the lag order, indicating that urbanization has a long-term and sustained driving effect on EG. The cumulative response coefficient of UR to EC also showed an upward trend, increasing from 0.089 in period 4 to 0.174 in period 10, but the increase was relatively slow. These results verify Hypothesis H4, indicating that urbanization, through mechanisms such as population agglomeration, industrial agglomeration, and infrastructure sharing, has a sustained positive impact on economic scale and energy demand. Furthermore, it can be observed that the cumulative effect of UR on GDP is significantly stronger than its cumulative effect on EC, indicating a certain degree of decoupling between EG and EC during the urbanization process, which supports sustainable urbanization.

**Table 10:** Cumulative impulse response of UR to GDP and EC.

Lag period	UR → GDP	UR → EC
1	0.042	0.031
2	0.087	0.058
3	0.121	0.076
4	0.152	0.089
5	0.186	0.104
6	0.221	0.104
7	0.256	0.133
8	0.294	0.147
9	0.339	0.160
10	0.387	0.160

This study empirically estimated the EKC hypothesis to test whether an inverted U-shaped relationship exists between EQ and economic development. Table 11 presents the estimation results of the EKC models. Model 1 only includes GDP and GDP², while models 2–4 gradually incorporate control variables such as UR and EC intensity. Model 5 uses fixed-effects estimation to control for individual heterogeneity. Table 11 shows that in all models, the GDP coefficient is significantly positively correlated, and the GDP² coefficient is significantly negatively correlated, all passing the 1% or 5% significance level test. This result demonstrated a clear inverted U-shaped relationship between EQ and economic development, validating the rationality of the Environmental KC hypothesis. Based on the estimation results of Model 5, the EKC inflection point is calculated to be approximately 2830 yuan. Combining the sample data, the province’s per capita GDP reached this inflection point around 2006. Before the inflection point, EG was accompanied by a decline in EQ. After the inflection point, EG and environmental improvement showed a synergistic development trend. This finding aligns with the theoretical expectations of the Environmental KC and also echoes the conclusion in impulse response analysis that the long-term impact of economic development on EQ tends to be insignificant. Regarding control variables, UR showed a significant positive correlation in Models 2 and 3, but the significance weakened after adding year-fixed effects, indicating that the impact of urbanization on EQ is moderated to some extent by the stage of economic development and policy factors. EC showed a significant positive correlation in all models, with a large coefficient, indicating that EC is a key factor affecting EQ. This is consistent with the Granger causality test result that EC represents the Granger cause of EQ.

**Table 11:** Empirical test results of EKC.

Variables	Model 1	Model 2	Model 3	Model 4	Model 5
GDP	1.324***	1.156***	0.987***	0.892***	0.763**
	(0.187)	(0.165)	(0.142)	(0.138)	(0.156)
GDP²	−0.089***	−0.076***	−0.064***	−0.057***	−0.048**
	(0.012)	(0.011)	(0.009)	(0.009)	(0.011)
UR	/	0.342**	0.287*	0.231	0.198
	/	0.342**	(0.151)	(0.149)	(0.162)
EC	/	/	0.418***	0.376***	0.352**
	/	/	(0.087)	(0.082)	(0.094)
Constant term	−2.156***	−1.987***	−1.654***	−1.432***	−1.208**
	(0.324)	(0.298)	(0.276)	(0.268)	(0.291)
Year fixed effect	No	No	No	Yes	Yes
Individual fixed effect	No	No	No	No	Yes
R²	0.412	0.448	0.486	0.521	0.547
Adjusted R^2^	0.398	0.448	0.467	0.498	0.512
F-statistic	28.46***	24.37***	22.19***	18.63***	15.42***
Observations	28.46***	152	152	152	152

Note: *, **, and *** indicate significance at the 1%, 5%, and 10% significance levels, respectively. The values in parentheses represent robust standard errors. A higher EQ value indicates worse EQ.

**Fig 12:**
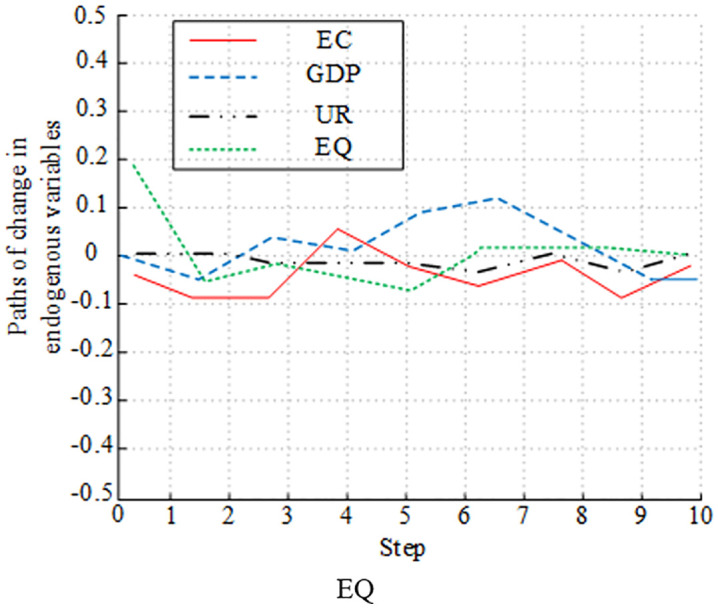
Plot of impulse response analysis between variables (EQ).

**Fig 13:**
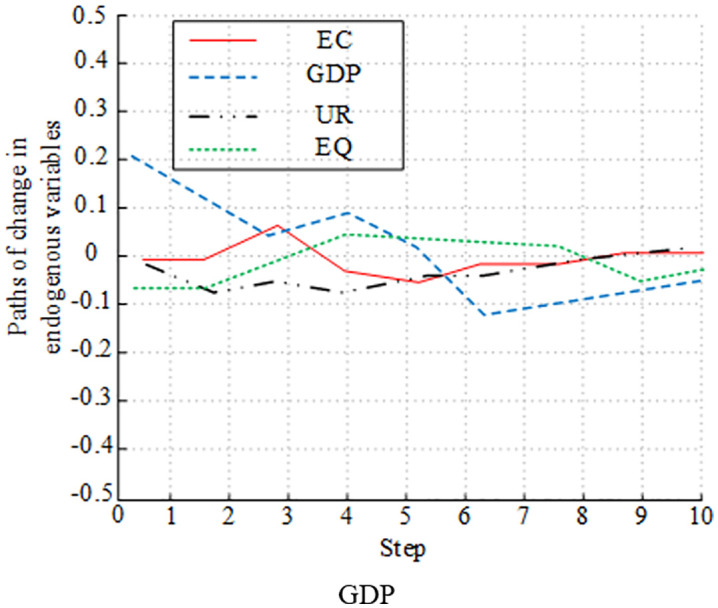
Plot of Impulse response analysis between variables (GDP).

**Fig 14:**
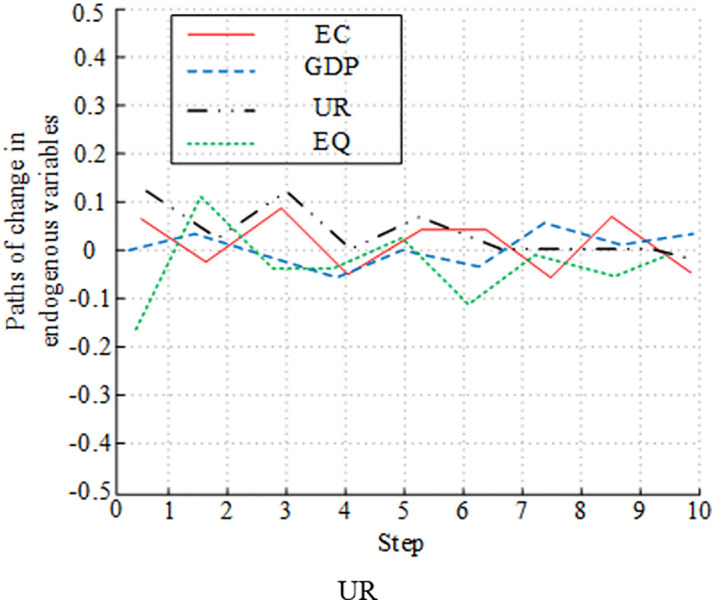
Plot of impulse response analysis between variables (UR).

**Fig 15:**
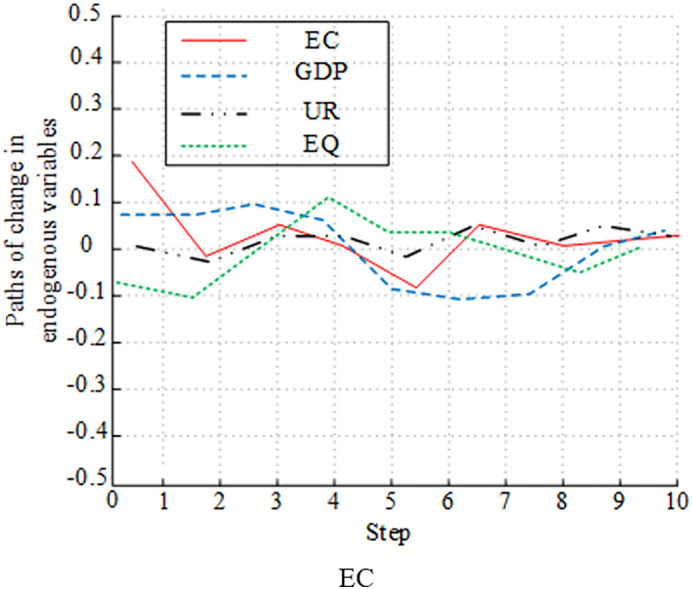
Plot of impulse response analysis between variables (EC).

Figs 12-15 presents the experimental results of analyzing the impulse effect of four variables on economic development and EC. In Fig 12, a positive shock to EC caused EQ to fluctuate negatively in the early stage. Then, it gradually recovered and returned to a steady state. The impact of a positive shock to GDP on EQ followed a similar trend to EC, initially negative and then recovering. The impact of UR on EQ was weak, with small overall fluctuations. EQ’s own response to shocks was significant in the early stage, but gradually weakened due to the influence of other variables in the later stage. In Fig 13, a positive shock to UR had a sustained positive promoting effect on GDP, and the impact was relatively significant. A positive shock to EC had only a weak positive impact on GDP in the short term and a weak long-term effect. The impact of EQ on GDP was not obvious. In the early stage, GDP’s response fluctuated significantly, and its explanatory power gradually weakened due to the influence of variables such as UR and EC in the later stage. Fig 14 shows that a positive shock to GDP significantly fluctuated UR in the short term, but had a driving effect in the long term. A positive shock to EC also had a fluctuating effect on UR. UR’s response to shocks was relatively weak. The impact of EQ on UR changed significantly in the early stage, and gradually stabilized in the later stage. Fig 15 shows the long-term impact of a positive GDP shock on EC. There was an initial rise, followed by a fall, and then another rise. The response remained positive in the long term. A positive EQ shock caused EC to gradually recover after an initial negative fluctuation. The impact of UR on EC showed relatively minor fluctuations and was not significant. While it fluctuated significantly in the early stages, it gradually stabilized in the later stages due to the influence of variables such as GDP and EQ.

**Fig 16:**
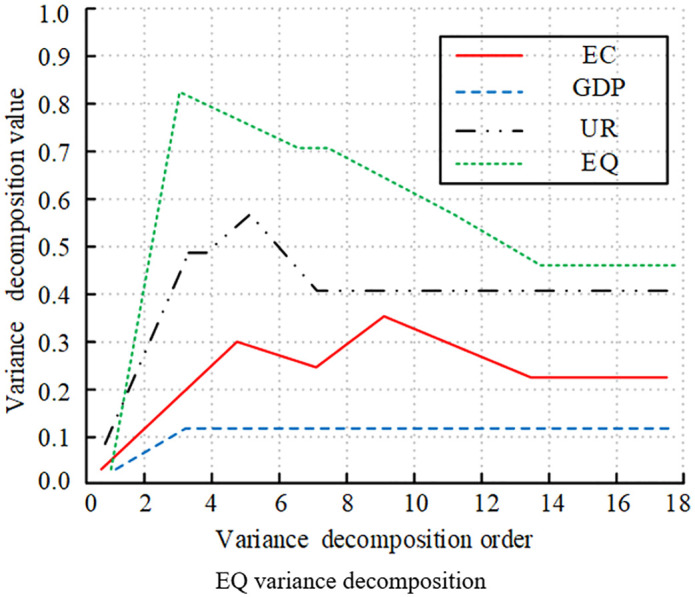
Decomposition of variance for different variables (EQ variance decomposition).

**Fig 17:**
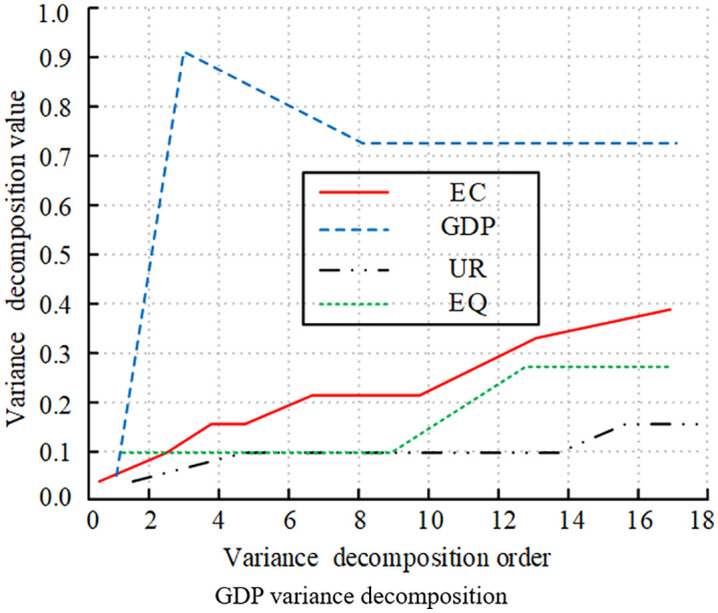
Decomposition of variance for different variables (GDP variance decomposition).

**Fig 18:**
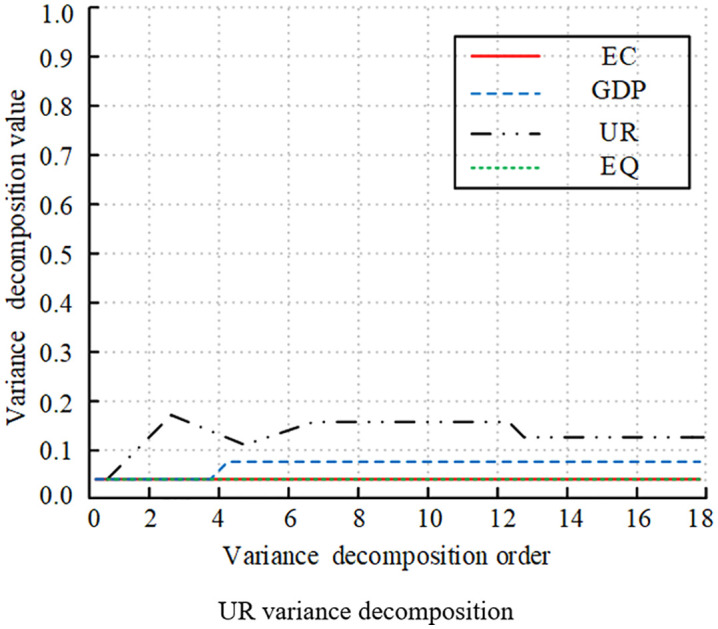
Decomposition of variance for different variables (UR variance decomposition).

**Fig 19:**
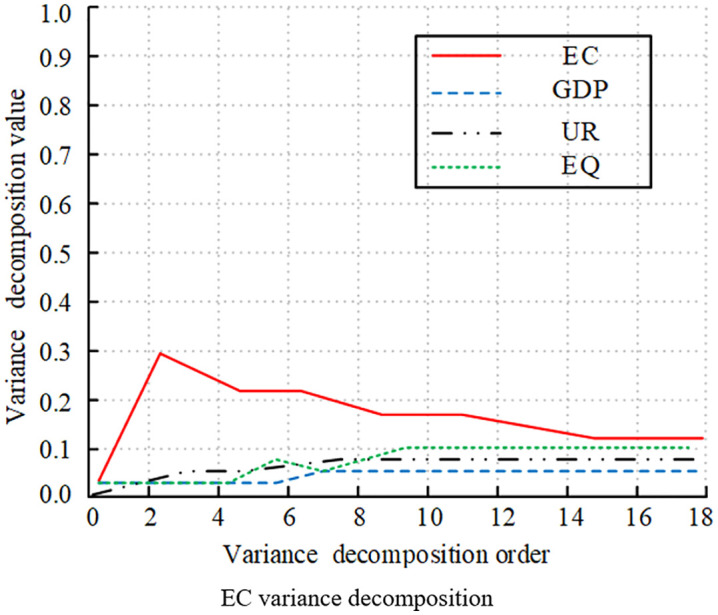
Decomposition of Variance for Different Variables (EC variance decomposition).

Figs 16-19 illustrates the statistical results of variance decomposition, highlighting the impact of variable shocks on variable volatility. In Fig 16 the contribution of EQ to its own volatility gradually increased in the early stages, peaking above 0.8, which was quite significant. It later declined rapidly due to the influence of EC and EG. This indicated that short-term EQ volatility was primarily self-explanatory, while long-term volatility was influenced by external variables. In Fig 17, GDP’s own contribution rose above 0.9 at order 4, then slowly declined and gradually stabilized above 0.7. The contributions of EC and EQ continue to rise, significantly impacting GDP volatility. UR’s contribution remained relatively small, with a weak impact on GDP volatility. Fig 18 shows that UR itself had a significant impact. It initially rose slowly and then stabilized around 0.15, with relatively small overall volatility. The impact of GDP on UR rose slowly and eventually stabilizes at 0.08. Meanwhile, the effects of EC and EQ remained consistently around 0.04, having no significant impact on UR volatility. Fig 19 shows that the EC shock significantly contributed to volatility, gradually increasing to a peak of 0.3 in the early stages, then slowly decreasing, and eventually stabilizing at around 0.18. The impacts of GDP, UR, and EQ on EC increased slowly as well.

**Fig 20:**
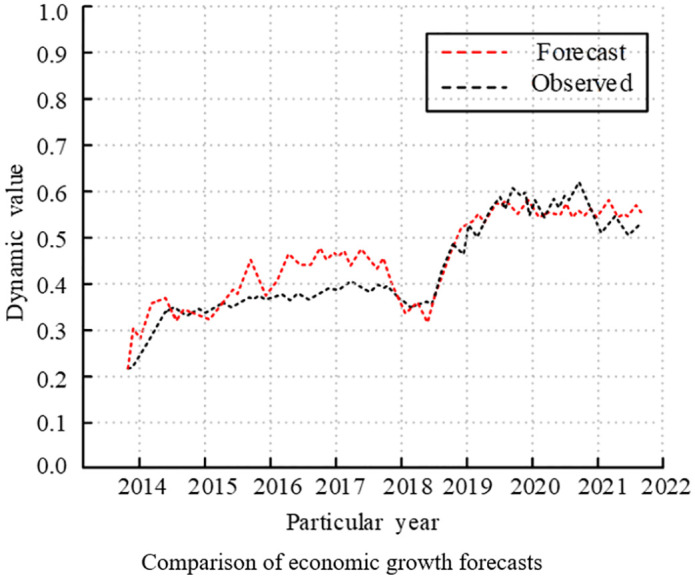
Comparison of dynamic prediction results analysis (Comparison of economic growth forecasts).

**Fig 21:**
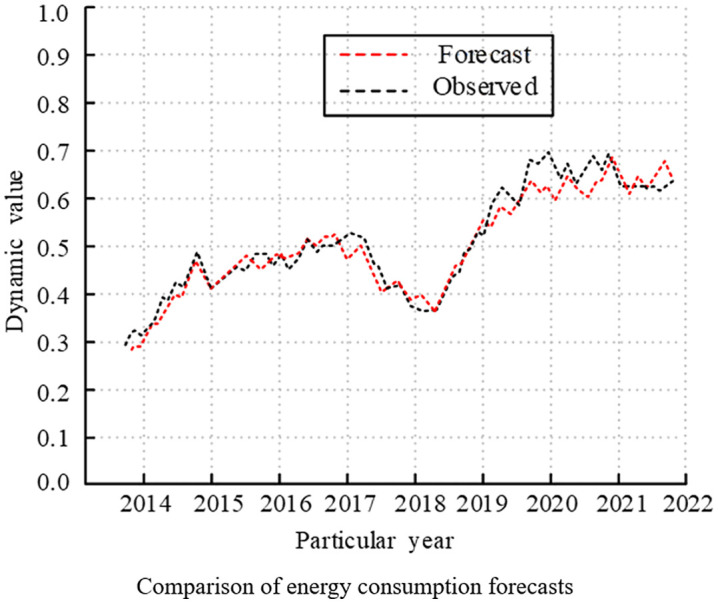
Comparison of dynamic prediction results analysis (Comparison of energy consumption forecasts).

Finally, comparing the predicted and observed values of the model for EG and EC, the experimental results are shown in Figs 20 and 21. The observed and anticipated values for the EG and EC variables were essentially in line with the trend of change, and the prediction results were extremely trustworthy. In summary, the improved VAR model designed by the study could reflect the economic reality.

## 4. Discussion

This study reveals the dynamic interaction mechanism among urbanization, EG, EC, and EQ. Granger causality tests show a bidirectional causal relationship between EG and EC (H1), EG is a Granger cause of EQ (H2), the impact of EG on EC exhibits a non-linear decay characteristic (H3), and urbanization has a continuous driving effect on both EG and EC (H4). These findings are consistent with theoretical expectations.

Theoretically, the bidirectional causal relationship between EG and EC is consistent with the basic logic of neoclassical growth theory. Energy, as an essential factor in the production process, directly drives economic output, while the income increase brought about by EG, in turn, increases the demand for energy. Recursive estimation results showed that the impulse response coefficient of GDP to EC decreased from 0.216 in 1985–2000 to 0.142 in 2001–2022, which verifies hypothesis H3, that the dependence of EG on EC shows a non-linear decreasing trend with changes in the development stage. This finding echoes the viewpoint proposed by Xing et al. [11], who argued that decoupling EG from EC can be achieved through structural adjustments and policy interventions. The cumulative response coefficient of UR to GDP increased from 0.152 in period 4 to 0.387 in period 10, while the cumulative response coefficient to EC increased from 0.089 to 0.174. It is worth noting that the cumulative effect of UR on GDP is significantly stronger than its cumulative effect on EC, indicating that EG and EC exhibit a certain degree of decoupling during the urbanization process.

The findings of this study are consistent with existing empirical studies, but also differ to some extent. Regarding the relationship between EG and EC, Rehman et al. found that EC and per capita GDP are positively correlated, which is consistent with the conclusion of a two-way causal relationship drawn in this study. However, Rehman et al. did not find the characteristics of the relationship changing over time, while this study identified this nonlinear change characteristic through time-segmented estimation. Regarding the relationship between EG and EQ, studies by Sadiq et al. [9] and Bui et al. [10] have shown that EG leads to greater environmental pressure, a finding verified by the Granger causality test in this study. However, impulse response analysis revealed that this effect gradually weakens in the long term, indicating that the time factor is important in the analysis of the relationship between the environment and the economy. Regarding the role of urbanization, studies by Abdi et al. [23] have shown that urbanization leads to a continuous increase in the ecological footprint, consistent with the conclusion in this study that UR has a sustained positive driving effect on EC. Furthermore, this study found that urbanization’s driving effect on EG is more significant than its impact on EC, providing more detailed practical data support for understanding the dual role of urbanization.

This study empirically tested the EKC model and found that the GDP coefficient is significantly positive, while the GDP² coefficient is significantly negative, demonstrating a clear inverted U-shaped relationship between EQ and economic development. Based on the estimation results of Model 5, the EKC inflection point is approximately 2830 yuan per capita GDP, a point reached by the province around 2006. This finding is consistent with the classic EKC theory and echoes the findings of Xing et al. [11]. The empirical results of EKC in this study and the conclusions drawn from impulse response analysis can corroborate each other. The impulse response analysis shows that the long-term impact of economic development on EQ will gradually become insignificant, indicating that the province has entered the development stage after the EKC inflection point, and EG and environmental improvement can achieve coordinated development. The analysis of control variables shows that EC intensity is significantly positive in all models, indicating that EC is still a key factor affecting EQ, which is highly consistent with the conclusions drawn by Raihan et al. [24].

Based on the above research conclusions, it can be found that the dependence of EG on EC will show a non-linear decline as the development stage changes. Policy formulation should consider the differences in different development stages. In the early stage of industrialization, the focus should be on improving energy efficiency, while in the later stage, the focus should be on promoting industrial structure transformation. Urbanization can continuously drive EG, but its impact on EC is relatively small, which provides policy space for sustainable urbanization. In the future, priority can be given to the development of green buildings and low-carbon transportation to optimize the spatial layout of cities. EC is a key factor affecting EQ. Governments need to strictly enforce environmental regulations, increase investment in environmental governance, and establish a dual control mechanism for total EC and intensity. The EKC inflection point has already appeared, indicating that EG and environmental improvement can be achieved simultaneously. Going forward, ecological civilization construction should be integrated into the entire process of economic development, and a green GDP accounting system should be established.

## 5. Conclusion

To explore the dynamic relationship between urban EG and EC and the influencing mechanism, the study took the VAR model as the technical basis, and introduces the GARCH model with the improved DE algorithm optimized in terms of time series data processing and parameter optimization. Based on the experimental results, the enhanced DE algorithm designed in the study demonstrated high population adaptability and stability. The ideal objective value converged at 0.09 with adequate population spacing. The adaptive and clustering improvement strategies enhanced the solution performance of the algorithm, and the optimal values of HV and IGD converged to 0.92 and 0.08, respectively. There were obvious Granger causality relationships between the four research variables selected in the study, among which economic development and EC directly affected the deterioration of EQ, with the significance levels lower than 0.1 and 0.05, respectively. The results of the impulse effect analysis also confirmed this view. In addition, the impulse effect results revealed that EC was not a long-term stable stimulus for EG, but EG needed the support of EC. The variance decomposition reflected the contribution of each variable to the generation of fluctuations in a given variable, with EG and EC generating the most significant impact of fluctuations. Comparison of predicted and observed results verified the credibility of the methodology used in the study. The improved VAR model designed in the study can realize the dynamic correlation analysis of urban EG and EC, which is helpful for the prediction of future development trends.

The study’s comprehensive analysis of the social, economic, energy, and environmental aspects yielded the following policy recommendations, which are designed to achieve sustainable economic development, continuously promote the urbanization process, and optimize the energy and industrial structure. First, priority must be given to the development of green buildings and low-carbon transport in urbanization. Moreover, policy support must be increased to promote the development of renewable energy, and urban planning must be strengthened to optimize the spatial layout of cities, thereby reducing unnecessary EC and land waste. Specifically, the construction of green buildings and low-carbon transport projects can be incentivized through the provision of tax incentives and financial subsidies, while supervision can be strengthened to ensure that the relevant policies are effectively implemented. Secondly, the implementation of policies aimed at enhancing energy efficiency, fostering green industries, and fortifying the regulatory framework of the energy market is imperative. This is to mitigate the adverse consequences of energy price volatilities on the economy and to ensure the continuity of stable EG. In the course of implementation, the rapid development of green industries can be promoted through the provision of technical support and financial support to improve the efficiency of energy use. It will also strengthen the transformation and upgrading of traditional high-energy-consuming industries and promote their transformation in the direction of low-carbon and environmental protection. Thirdly, the government should strictly enforce environmental regulations and standards, promote environmentally friendly production and consumption patterns, and increase investment in environmental governance. When implemented, the enforcement of environmental protection laws and regulations should be improved to ensure that EQ is effectively improved. Meanwhile, efforts must be made to enhance public awareness of environmental protection through publicity and educational initiatives. The concept of green consumption should be promoted to foster a favorable atmosphere in which the entire society can engage in environmental protection.

However, EG and EC involve many factors and dimensions. This study only selects four variables for analysis: urbanization, economic development, EC, and EQ. It do not separately include key direct driving factors such as industrial development level, production technology mode, and industrial structure proportion. Furthermore, the proportion of the urban population is used to measure urbanization, but this failed to fully reflect the indirect transmission mechanism to EC. Future research needs to supplement these variables and control them, and further refine the analysis of driving paths. In addition, future research can introduce more theories from economics, sociology, and environmental science. With the help of advanced artificial intelligence technology, the dynamic relationship between urban EG and EC, as well as the influence mechanism, can be explored more deeply.
